# Efficacy and safety of Iguratimod as an add-on therapy for refractory lupus nephritis: A preliminary investigational study

**DOI:** 10.3389/fimmu.2023.1062919

**Published:** 2023-03-08

**Authors:** Qingran Yan, Mei Zhang, Fang Du, Yuening Kang, Ping Ye, Qianqian Li, Bei Liu, Min Dai, Chunde Bao

**Affiliations:** ^1^ Department of Rheumatology, Ren Ji Hospital, Shanghai Jiao Tong University School of Medicine, Shanghai, China; ^2^ Department of Nephrology and Rheumatology, The First Affiliated Hospital of Anhui Medical University, Hefei, China; ^3^ Department of Nephrology and Rheumatology, Anhui Public Health Clinical Center, Hefei, China; ^4^ The Seventh Affiliated Hospital, Sun Yat-Sen University, Shenzhen, Guangdong, China

**Keywords:** IGU, refractory lupus nephritis, combinational therapy, add-on, prospective study

## Abstract

**Objectives:**

IGU (IGU), a novel immunomodulatory agent for rheumatoid arthritis, has been shown to be effective and safe as monotherapy in a small population with refractory lupus nephritis (LN). The aim of this prospective study was to evaluate the efficacy and safety of IGU as an add-on therapy in patients with refractory LN in the context of clinical practice.

**Methods:**

This is a single-arm observational study. We have enrolled LN patients since 2019 at Renji Hospital. All participants should have recurrent or refractory LN with at least one immunosuppressant (IS) and have a baseline urine protein/creatinine ratio (UPCR) >1.0. After enrollment, we added IGU (25 mg twice daily) to one of their previous immunosuppressants (IS) without increasing the dose of steroids. The primary outcome was the complete renal response (CRR) in the 6th month. UPCR decrease of over 50% was defined as partial response (PR). Extended follow-up was performed after the initial 6 months.

**Results:**

We enrolled 26 eligible participants. 11/26 patients had chronic kidney disease (CKD) stage 2/3 at the baseline. The IS combined with IGU included mycophenolate mofetil, tacrolimus, and cyclosporin A. No IS change was allowed. 80.7% of patients had baseline steroids less than 0.5mg/kg daily and there was no steroids escalation during the IGU treatment. The CRR rate was 42.3% (11/26) at month 6. With a median follow-up of 52 weeks (range: 23-116 weeks), the CRR rate at the last visit was 50% (13/26) and 73.1% (19/26) of patients had UPCR decrease of over 50%. Six patients withdrew, three for no response and three for renal flare after initial CRR. One patient had an estimated glomerular filtration rate worsening of over 20% and was classified as renal flare. Three mild to moderate adverse events were recorded.

**Conclusions:**

Our investigation merits further investigation in IGU as a potentially tolerable component of combination therapy for refractory LN.

## 1 Introduction

Systemic lupus erythematosus (SLE) is an autoimmune disease that can involve multiple organs or systems (1–3). Lupus nephritis (LN) is associated with high mortality and morbidity rates. Over recent decades, substantial progress has been made in developing immunosuppressant agents and biologic therapies (4). However, a significant proportion of patients either do not respond to first-line immunosuppressive drugs or quickly relapse after initial remission. Approximately 10% of patients with LN will experience continued worsening of renal function and go on to develop end-stage renal disease (5).

To treat refractory LN, the European League Against Rheumatism (EULAR) recommendation suggests a switch either from cyclophosphamide (CYC) to mycophenolate mofetil (MMF) or vice versa. Moreover, combinational therapy is a common strategy in a series of observational studies for refractory LN (6–8). And recently, multi-targeted therapy such as MMF combined with a calcineurin inhibitor (CNI) has been recommended (9–12). The combination therapy of a study agent and a conventional immunosuppressant (IS) is a popular design of present trials for LN: such as trials for belimumab (13), voclosporin (14), obinutuzumab (15), and anifrolumab (16). However, in the context of clinical practice, the efficacy and safety of an agent in combinational treatment need a more cautious interpretation without a control arm.

IGU (IGU), a new immunomodulatory drug, has been approved for treating rheumatoid arthritis (RA) in northeast Asia. According to data from RA clinical trials in Japan and China, IGU is superior to placebo and non-inferior to methotrexate and sulfasalazine (17–20). Mechanically, as a disease-modifying drug for RA, IGU has been discovered to reduce inflammation *via* the nuclear factor-κB (NF-κB). IGU interferes with TNF-α-induced translocation of NF-κB and suppresses TNF-α-induced production of IL-6, IL-8, and monocyte chemoattractant protein 1 (MCP1) (21, 22). Besides, IGU selectively disturbs Act1-TRAF5 connections and TRAF5-Ikki interactions, interrupting IL-17 signaling (23). IGU inhibits macrophage migration inhibitory factor (MIF) tautomerase activity and prevents MIF-induced proinflammatory effects, therefore sparing steroids (24). COX-2 activity and transcription are both inhibited by IGU (25).

IGU has shown efficacy in LN-like disease of MRL/lpr mice (26). Interestingly, we further found IGU interference human B cell terminal differentiation *via* PKC/EGR1 axis (27). Recently, we reported 13/14 patients with refractory LN responded to IGU monotherapy at week 24 (28). In this study, we aimed to explore the efficacy and safety of IGU as a component of combination therapy for refractory LN. For this, we applied an add-on design, which we believe is effective for elucidating.

## 2 Materials and methods

### 2.1 Study design

We have screened the medical records from Renji Hospital since 2019. All participants should have recurrent or refractory LN with at least one IS and have a baseline urine protein/creatinine ratio (UPCR) >1.0. Failure was defined as no remission (not achieving complete renal response (CRR) or having UPCR decrease over 50%, see below in outcomes) on one agent for at least 6 months. Once a patient had been enrolled, he or she was prescribed oral IGU at a dose of 25mg twice daily in addition to one of the IS that the patient previously used with an insufficient response (failure or flare). Meanwhile, the patients continued other medications, such as steroids, anti-malaria drugs, or angiotensin converting enzyme/receptor inhibitor (ACEI/ARB), without dose adjustment. All patients gave written informed consent. The study was approved by the Ethics Committee of Renji Hospital, Shanghai, China.

### 2.2 Outcomes

The complete renal response (CRR) (14) at 6 months was used as the primary outcome, i.e., UPCR ≤0.5 with estimated glomerular filtration rate (eGFR) ≥60 mL/min/1·73 m^2^ or no confirmed decrease from baseline in eGFR of >20% (14). UPCR decrease of over 50% was assessed as partial response (PR) at each visit as a key supplementary treatment target (9), especially for refractory LN. After 6 months, the renal response would be continuously assessed.

Other outcomes evaluated included renal flares, extra-renal flares, and safety. A renal flare was defined according to Joint European League Against Rheumatism and European Renal Association–European Dialysis and Transplant Association (EULAR/ERA-EDTA) recommendations for the management of adult and pediatric lupus nephritis (29). An extra-renal flare was defined as the presence of manifestations that could be attributed to SLE that required high-dose steroids. Any need for treatment escalation over one week, including daily prednisone ≥ 1mg/kg or add/switch to another IS/targeted therapy would be counted as treatment failure and lead to termination of the follow-up.

Grading of the severity of adverse events was carried out using the National Cancer Institute Common Terminology Criteria for Adverse Events (CTCAE), version 4.03 (grade scale 0–5).

## 3 Statistics

Baseline clinical characteristics of the study participants were summarized using medians with ranges for continuous variables and proportions for categorical outcomes. Mann-Whitney test was used for group comparison of continuous variables. Fisher exact chi-squared test or likelihood-ratio test was used for group comparison of categorical outcomes. The alternative hypothesis was accepted at a statistical significance level of P<0.05 on all applied statistical tests. Analyses were conducted using IBM SPSS Statistics 25.0. Transition plots for renal outcomes were performed using R language software (Version R 4.2.1).

## 4 Results

### 4.1 Characteristics of patients

From 2019 to 2022, we screened 32 patients for this study. Five of these patients failed to meet the inclusion criteria and one patient withdrew her consent. A total of 26 participants were eligible and enrolled. 24/26 participants were female. The median disease duration of LN was 5 years (range: 0.8-19 years). 80.8% (21/26) of the nephritis had been confirmed by biopsy (WHO class III/IV/V) (30). Major clinical characteristics are shown in **Table 1**.

**Table 1:** Baseline characters and outcomes of the study population.

Patient No.	Age	Sex	LN duration (yrs)	Pathology	Previous treatment	Baseline steroids ≥0.5mg/kg	IGU combination	Baseline CKD stage	Baseline UPCR	Follow-up (w)	Outcome at the last visit
1	28	F	9	IV	CTX(NR)→MMF(NR)→TAC(NR)→LEF(NR)	No	MMF 1.5g	1	4.98	68	NR*
2	29	F	4	IV+V	AZA(NR)→CTX(NR)→TAC(NR)→MMF+RTX(NR)	No	MMF 1.5g	2	4.90	64	PR
3	33	F	5	IV+V	MMF(NR)→CTX(NR)→TAC(NR)	No	MMF 1.5g	2	2.89	24	NR*
4	33	F	5	IV+V	MMF(NR)	No	MMF 1.5g	3	1.13	51	PR
5	27	F	4	IV	MMF(NR)→CTX(NR)→AZA(NR)	Yes	MMF 1.5g	1	2.92	48	CRR
6	39	F	2	V	AZA(NR)→TAC(NR)	No	TAC 1.5mg	1	2.18	24	PR
7	27	F	1	III	MMF(NR)→CTX(CRR)→TAC(R)	Yes	TAC 1mg	1	1.49	26	CRR
8	51	F	19	IV+V	CTX(NR)→AZA(R)→MMF(NR)→LEF(NR)→TAC(R)→MMF+TAC(NR)→TAC+AZA+THA(NR)→RTX(NR)→AZA(R)→MMF(R)	Yes	MMF 0.75g	2	5.73	52	Relapse*
9	32	F	0.8	N/A	TAC (R)	No	TAC 1mg	1	1.46	54	CRR
10	30	F	18	IV	MMF(R)	No	MMF 1.0g	1	1.58	116	CRR
11	34	F	1	N/A	MMF(R)	No	MMF 1.5g	1	1.62	48	CRR
12	59	F	1.5	V	AZA(NR)→MMF(NR)	No	MMF 1.0g	1	2.38	45	CRR
13	39	F	11	V	CsA(NR)→MMF(NR)→TAC(NR)	Yes	MMF 1.5g	1	4.03	44	NR
14	33	F	20	IV+V	CTX(R)→MMF(NR)	Yes	MMF 0.75g	2	4.64	96	PR
15	28	F	5	N/A	MMF(R)→TAC(R)	No	TAC 2mg	1	4.45	48	Relapse*
16	61	F	12	IV	MMF(NR)→TAC(NR)→CTX(R)	No	MMF 1.5g	2	1.28	24	Relapse*
17	26	F	8	V	CTX(NR)→MMF(NR)→TAC(NR)	No	MMF 1.5g	1	4.78	79	CRR
18	39	F	2	IV	CTX(NR)→MMF(NR)	No	MMF 1.5g	2	2.77	52	CRR
19	29	F	5	IV	CTX(NR)→MMF(NR)→TAC(NR)	No	MMF 2.0g	1	2.98	68	PR
20	35	F	10	III	CTX→AZA(R)	No	AZA 50mg	1	2.93	41	CRR
21	50	M	14	IV+V	CTX(CR)→AZA(R)→CTX(PR)→CsA(NR)→MMF(NR)→CTX(NR)→TAC(R)→LEF(R)→TAC(NR)	No	MMF 1.0g	2	2.66	77	PR
22	33	F	12	III+V	CTX(CRR)→MMF(R)→TAC(NR)	No	MMF 1.0g	2	3.09	23	CRR
23	31	F	4	N/A	MMF(NR)→CsA(NR)→TAC(NR)→CTX(NR)→LEF(NR)	No	CsA 50mg	2	1.56	53	CRR
24	40	M	12	II	MMF(NR)→TAC(NR)	No	MMF 1.5g	1	2.84	67	CRR
25	29	F	14	V	CTX(NR)→AZA(NR)→TAC(NR)→MMF(NR)	No	MMF 1.5g	2	17.76	48	NR*
26	37	F	14	V	AZA(CRR)→TAC (NR)→AZA(NR)	No	AZA 100mg	1	1.924	102	CRR

*Withdraw with modified IS regimen or escalation of steroids.

AZA, azathioprine; CKD, chronic kidney disease; CRR, complete renal response; CsA, cyclosporin A; CTX, cyclophosphamide; IS, immunosuppressants; LEF, leflunomide; LN, lupus nephritis; MMF, mycophenolate mofetil; NR, no response; PR, partial response; R, relapse; RTX, rituximab; TAC, tacrolimus; UPCR, urine protein/creatinine ratio.

The arrows in the previous treatment column represent treatment switch.

The median amount of baseline UPCR was 2.808 (range: 1.13-17.76). None of the patients had detectable evidence of active extra-renal organ involvement, probably because of long-term steroid/immunosuppressive therapy and long disease duration (31). At enrollment, the median eGFR was 97 ml/minute/1.73 m^2^ (range: 52-132), ten patients had chronic kidney disease (CKD) stage 2, and one was in stage 3. 25/26 patients had renal pathology results, most of which were performed at the diagnosis of LN. One patient had too few glomeruli to calculate a reliable acute or chronic index (AI/CI). Among the 24 left patients, the median AI of renal pathology was 8.00 (IQR: 6.25-11.00), and the median CI was 3.00 (IQR 3.00-5.75). The IS combined with IGU included mycophenolate mofetil, tacrolimus, and cyclosporin A. 80.7% (21/26) of patients had steroids less than 0.5mg/kg daily and the steroids dose of the other five patients was in the range of 0.5mg/kg - 1.0mg/kg daily at baseline. (**Table 1**)

The baseline median anti-dsDNA antibody level was 37.47 IU/mL (range: 6.4-100) by Farr method. The baseline median serum C3 was 0.73g/L (range: 0.36-1.20) by quantitative turbidimetric assay.

### 4.2 Efficacy outcomes

**Figure 1:**
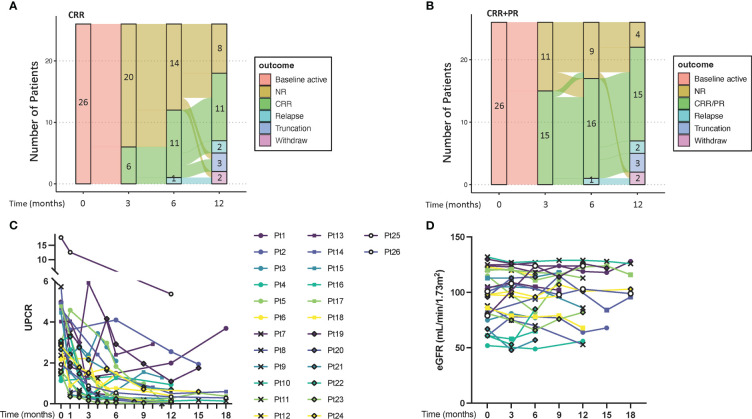
The transition and renal outcomes of the patients. **(A, B)**, transition plots for renal outcomes defined by CRR and CRR+PR, respectively. **(C)** UPCR of each patient. **(D)** eGFR of each patient. CRR, complete renal response; PR, partial response; eGFR, estimated glomerular filtration rate; NR, no response; UPCR, urine protein/creatinine ratio.

The CRR rate was 42.3% (11/26) at month 6. With a median follow-up of 52 weeks (range: 23-116 weeks), the CRR rate at the last visit was 50% (13/26) and 76.9% (20/26) of patients had a UPCR decrease of over 50%. (**Table 1**) The renal outcome transition from the baseline to the 12th month is illustrated in **Figures 1A, B**. Five patients (Patient 2, 4, 6, 14, and 21) did not achieve response up to 12 months yet had decreased UPCR >50% of baseline, so they were still with IGU treatment. (**Figure 1C**) Three patients developed renal relapse and exited the follow-up. Of these three, one patient (Patient 8) had an estimated glomerular filtration rate (eGFR) worsening of over 20%, despite of remission in proteinuria. Most patients kept eGFR stable. (**Figure 1D**). No steroids escalation was recorded during the IGU treatment. 18 patients experienced at least one reduction of steroids during follow-up. At the last visit, 24/26 patients had steroids ≤ 10mg/d (calculated as prednisone).

### 4.3 Safety profile

Two mild to moderate adverse events were recorded. One was leukocytopenia and another was an elevation of alanine aminotransferase. Both events were transient and recovered after symptomatic treatment. Among the five patients receiving the combination of IGU and CNIs, who had more concern for renal function (32), four patients in CKD 1 had eGFR stable and one patient with baseline CKD 2 (Patient 23) experienced a transient eGFR worsening and recovered automatically in the 12th month. Another patient receiving MMF+IGU (Patient 8), had slowly worsened eGFR with a complete remission in proteinuria. Given the decreasing manner and the increase in anti-dsDNA antibody from 6.4IU/L to 46.4IU/L, we classified this patient as a renal flare. No side effects lead to treatment stopping or withdrawal.

## 5 Discussion

In this study, we showed for the first time the feasibility of IGU as a component of combinational therapy for LN management. Previously we showed IGU monotherapy for refractory LN with a 92.3% (12/13) response rate at week 24 (28). The 6-month response rate was 46.2% (12/26) in this study, as the response criteria changed from traditional partial/complete response (11, 28, 33) to CRR and as a higher proportion of CKD in eligible patients (42.3% vs 8.3%).

We had 23 patients eligible for a 12-month analysis, 47.8% (11/23) of whom had CRR and 65.2% (15/23) had CRR+PR. In a recent randomized controlled trial of refractory LN, a combination of three agents, cyclophosphamide, rituximab, and belimumab, achieved 38% CRR and 52% CRR+PR at week 48 (34). In another small observational cohort of refractory lupus, 67% of twelve LN patients had CRR with combinational therapy of rituximab and belimumab (35). Despite different renal remission definitions, the response rate of our study is comparable to those previously reported in observational studies on several agents of refractory LN, including calcineurin inhibitors (9, 10), rituximab (6–8), and stem cell transplantation (25).

The common conventional IS for LN treatment (9), including MMF, CNIs, and azathioprine, were involved in this study. There was no observable discrepancy among these combinations, implying IGU is a versatile component. Of note, all the IS in this study were required to have insufficient response prior to IGU treatment. Moreover, most patients (84.6%) had steroids less than 0.5mg/kg and no patients escalated steroids during IGU treatment. Therefore, the efficacy of IGU could be effectively assessed under these circumstances, which also made the results of the study compelling and reliable despite the absence of a control arm.

Given the missing data, we did not perform further analysis on serum C3 or anti-dsDNA. Other subgroup comparisons between CRR and non-CRR patients showed some trends but not significantly, probably due to the limited number of patients, including sex, LN duration, renal pathology type, previous IS number, as well as baseline UPCR, and the stage of CKD (**Supplementary Figure**).

Recently, several new treatments, such as belimumab, voclosporin, obinutuzumab, and anifrolumab, have achieved positive primary endpoints or key secondary endpoints in phase II or phase III trials for general LN. These agents represent the current target of LN treatment, including B cell (obinutuzumab and belimumab), T cell (voclosporin), and innate immunity (anifrolumab). However, the CRR in these clinical trials is still far from satisfactory. The 1-year CRR is 35%-41% (13–16), with a conventional IS background treatment in each trial. With a composite effect on all the targets above, IGU inhibits B cell termination differentiation (27), NF-κB and IL-17 signaling in T cells (23, 36), and macrophage activator MIF (24). These features make IGU a competitive candidate for general LN treatment.

Our study had several limitations. 1) The major limitation of the current study is the small sample size with only 26 cases, partially because of the difficulty in collecting refractory LN patients, which is even true for a multicentered clinical trial (34). 2) This study did not contain a control arm. To minimize this shortness, we carefully controlled every confounding factor that might interfere with interpreting the results. The patients needed not only to have active baseline renal manifestations but to meet the criteria for refractory. No steroids or IS escalation over one week was allowed during follow-up. With these, we believe that the results show some value of IGU in this add-on design for LN treatment. 3) Although with a common difficulty for LN management in repeated renal biopsy (37), more differential diagnoses could be done, such as a repeated renal biopsy at the baseline of IGU use to rule out podocytopathy. 4) This study only enrolled patients from East Asia, an ethnic origin in which lupus is less severe as compared to other groups (38), therefore the results cannot be generalized to other patient such as Caucasians or Afro-Americans. 5) The median follow-up time was 52 weeks, which might miss the long-term outcome. To overcome these limitations, we are performing a randomized controlled clinical trial to compare the efficacy of IGU in the induction therapy of active LN with a longer follow-up period (NCT02936375).

## 6 Conclusion

Our findings imply the potential feasibility to explore IGU as a component of combination therapy for LN.

## Data availability statement

The data analyzed in this study is subject to the following licenses/restrictions: The raw data are internal data of Renji Hospital of Shanghai Jiaotong University School of Medicine and are not available to the public. Requests to access these datasets should be directed to QY (yanqingran@renji.com).

## Ethics statement

The study was approved by the Ethics Committee of Renji Hospital, Shanghai, China. The patients/participants provided their written informed consent to participate in this study.

## Author contributions

QY, MZ, and FD carried out the study with support from YK, PY, QL, and BL. CB and MD supervised the project. QY wrote the manuscript. All authors discussed the results and contributed to the final manuscript. All authors read and approved the final manuscript. All authors contributed to the article and approved the submitted version.

## Supplementary material

Supplementary Figure Subgroup analysis between CRR and non-CRR patients on age, sex, LN duration, renal pathology type, previous IS number, as well as baseline UPCR, and the stage of CKD. None of these statistics are significant. CRR, complete renal response; UPCR, urine protein/creatinine ratio; CKD, chronic kidney disease; LN, lupus nephritis; IS, immunosuppressants.
