# Supplementary material for: Efficacy and safety of Iguratimod as an add-on therapy for refractory lupus nephritis: A preliminary investigational study
Source: Front Immunol. 2023 Mar 8;14:1062919. doi: 10.3389/fimmu.2023.1062919 (PMC10030952; doi:10.3389/fimmu.2023.1062919)
Supplement: Supplementary Figure — Subgroup analysis between CRR and non-CRR patients on age, sex, LN duration, renal pathology type, previous IS number, as well as baseline UPCR, and the stage of CKD. None of these statistics are significant. CRR, complete renal response; UPCR, urine protein/creatinine ratio; CKD, chronic kidney disease; LN, lupus nephritis; IS, immunosuppressants. [file Image_1.pdf]

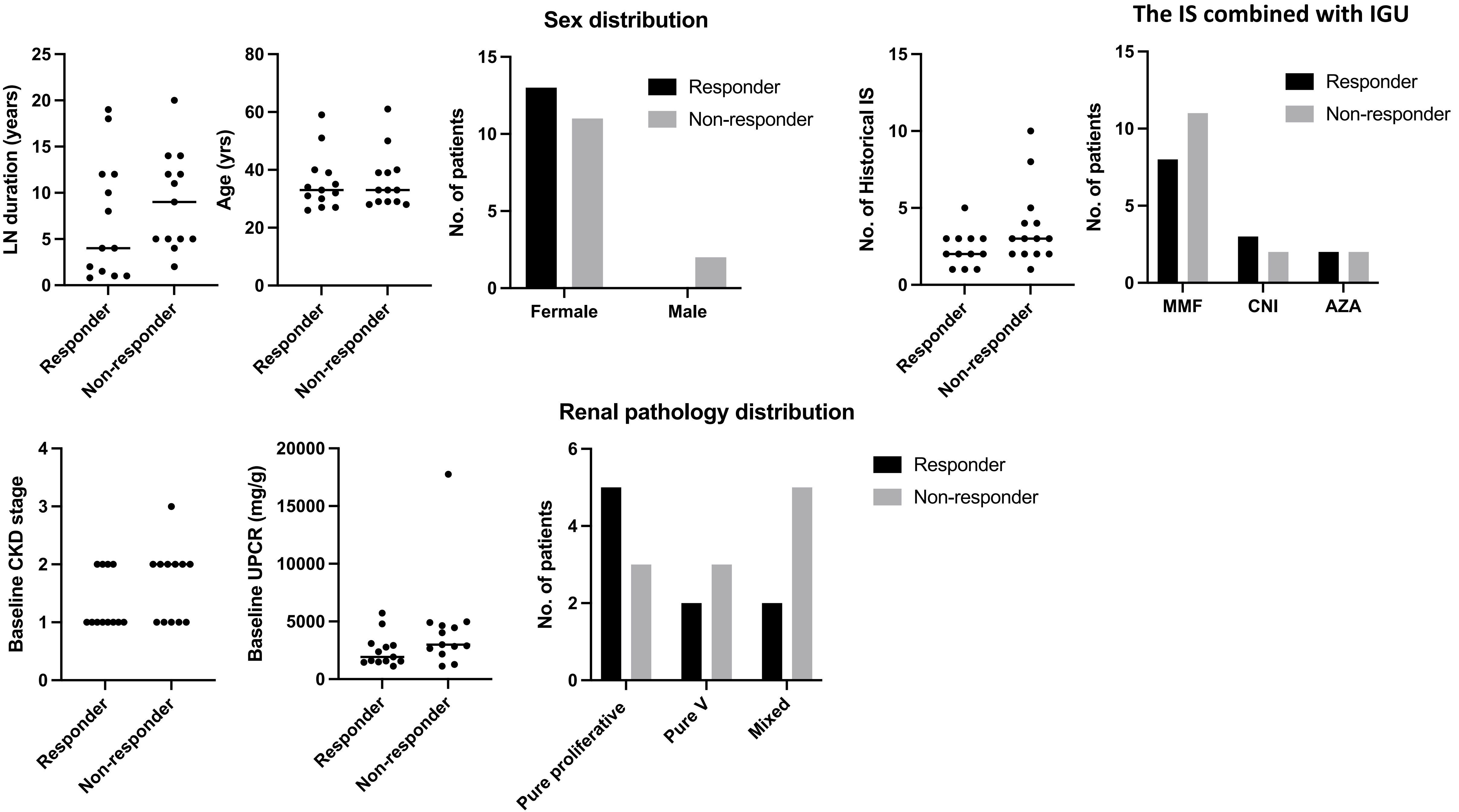

Sex distribution
The IS combined with IGU
Renal pathology distribution
LN duration (years)
Age (yrs)
No. of patients
No. of Historical IS
Baseline CKD stage
Baseline UPCR (mg/g)
Responder
Non-responder
Fermale
Male
MMF
CNI
AZA
Pure proliferative
Pure V
Mixed
0
5
10
15
20
25
40
60
80
1
2
3
4
5000
10000
15000
20000
6
